# Medical Phase of the Tonsil-Adenoid Clinic

**Published:** 1920-11

**Authors:** Albert D. Kaiser


### MEDICAL PHASE OF THE TONSIL-ADENOID CLINIC

BY ALBERT D. KAISER, M.D.

Children with abnormal tonsils and adenoids in many instances show other evidences of disease or deformity. Such conditions as discharging ears, enlarged glands of the neck, and evidences of chronic heart disease have been frequent occurrences among these children. It was deemed necessary to know all these facts not only to determine if it was safe to subject the child to an operation, but also to have a complete record of the child's physical status at the time of operation.

Upon admission to the dispensary the child was weighed and measured. A complete physical examination was made and a full record was kept on the physical examination card. Particular emphasis was placed on defects that may bear any relation to diseased tonsils, or develop mental defects due to obstructive adenoids. In these routine examinations a number of children were found in the early stages of an acute infection. Such children were immediately isolated and operation deferred. Inasmuch as these children were under the supervision of the dispensary only a short time, steps could not be taken, except for dental care, to direct treatment for any of the defects found. In many of such cases the patients already were under careful medical supervision, and if not, the doctor or agency reporting the case to the clinic was informed of the defect found, so that immediate steps could be taken to remedy the trouble or at least to keep the child under observation.

The purpose of this physical examination was obvious, for without it future examination would be of little value. It was planned to examine these children every six months after operation and record the findings. In some instances the second examination may be final, but enough subsequent examinations are advised to demonstrate whether or not the operation has benefited the child.

Experience has shown that quite a number of children present symptoms of tonsil and adenoid disturbances, and yet on physical examination, except for oral examination, appear normal. In order to correlate these complaints, the questionnaires were devised. The prevalence of developmental defects as evidenced by slow progress in school and misbehavior can not be ascertained by physical examination alone. The questionnaires, however, covered these facts.

The relationship of diseased tonsils to various infections and nutritional diseases is an accepted fact, but there is a large group of children where the pathology is not clearly known. Statistics on a large group are not available to prove that removal of tonsils and adenoids benefits this class. It is hoped that frequent examinations of these children will be particularly helpful in measuring and determining the exact value of the operation.

The medical examination in connection with the clinic had the following aims:

1. To guard against any surgical mishap by detection of serious defects which would endanger the child in case of operation;

2. To procure an accurate history of complaints presumably related to diseased tonsils and adenoids;

3. To make a physical examination bringing out any other remedial defects;

4. To refer to the proper agency for treatment if any other defect was ascertained;

5. To examine and question the children every six months to determine if the operation has done what was hoped for.

